# Bisphenol A Alters n-6 Fatty Acid Composition and Decreases Antioxidant Enzyme Levels in Rat Testes: A LC-QTOF-Based Metabolomics Study

**DOI:** 10.1371/journal.pone.0044754

**Published:** 2012-09-14

**Authors:** Minjian Chen, Bin Xu, Wenliang Ji, Shanlei Qiao, Nan Hu, Yanhui Hu, Wei Wu, Lianglin Qiu, Ruyang Zhang, Yubang Wang, Shoulin Wang, Zuomin Zhou, Yankai Xia, Xinru Wang

**Affiliations:** 1 State Key Laboratory of Reproductive Medicine, Institute of Toxicology, Nanjing Medical University, Nanjing, China; 2 Key Laboratory of Modern Toxicology of Ministry of Education, School of Public Health, Nanjing Medical University, Nanjing, China; 3 Jiangsu Provincial Center for Disease Control and Prevention, Nanjing, China; 4 Shanghai Office, Bruker Daltonics Inc., Shanghai, China; 5 Department of Epidemiology and Biostatistics, School of Public Health, Nanjing Medical University, Nanjing, China; 6 Safety Assessment and Research Center for Drug, Pesticide, and Veterinary Drug of Jiangsu Province, School of Public Health, Nanjing Medical University, Nanjing, China; Clermont Université, France

## Abstract

**Background:**

Male reproductive toxicity induced by exposure to bisphenol A (BPA) has been widely reported. The testes have proven to be a major target organ of BPA toxicity, so studying testicular metabolite variation holds promise for the discovery of mechanisms linked to the toxic effects of BPA on reproduction.

**Methodology/Principal Findings:**

Male Sprague-Dawley rats were orally administered doses of BPA at the levels of 0, 50 mg/kg/d for 8 weeks. We used an unbiased liquid chromatography-quadrupole time-of-flight (LC-QTOF)-based metabolomics approach to discover, identify, and analyze the variation of testicular metabolites. Two n-6 fatty acids, linoleic acid (LA) and arachidonic acid (AA) were identified as potential testicular biomarkers. Decreased levels of LA and increased levels of AA as well as AA/LA ratio were observed in the testes of the exposed group. According to these suggestions, testicular antioxidant enzyme levels were detected. Testicular superoxide dismutase (SOD) declined significantly in the exposed group compared with that in the non-exposed group, and the glutathione peroxidase (GSH-Px) as well as catalase (CAT) also showed a decreasing trend in BPA treated group.

**Conclusions/Significance:**

BPA caused testicular n-6 fatty acid composition variation and decreased antioxidant enzyme levels. This study emphasizes that metabolomics brings the promise of biomarkers identification for the discovery of mechanisms underlying reproductive toxicity.

## Introduction

Bisphenol A (BPA) is a chemical with one of the highest volume of production in the world, and in the U.S. the volume of BPA was estimated to be 2.4 billion pounds in 2007 [1]. BPA is used to produce polycarbonate and epoxy resins, which are used in baby bottles, lunch boxes as food and beverage packaging materials as well as dental sealants [2], [3]. Because of the extensive use of BPA, the general population may be exposed to BPA via inhalational, dermal and oral contact through foods and beverages as well as air, drinking water, dust, soil and personal care products [4]. In the 2003–2008 National Health and Nutrition Examination Survey (NHANES), the Centers for Disease Control and Prevention reported that BPA was detected in 92.6% of the persons in the United States [5].

BPA is one of many endocrine disrupting compounds (EDCs) and its health risk has aroused public concern recently [3]. Population based studies showed that BPA exposure is related to male reproductive abnormalities [6], [7]. Furthermore, the results of *in vitro* and *in vivo* studies have clearly demonstrated that exposure to BPA is a causal factor of spermatogenesis impairment [8], [9].

Metabolomics has been proven to be a useful tool in the discovery of new biomarkers for mechanisms of toxicity [10], [11]. To date, the study which focuses on BPA reproductive toxicity using a metabolomic approach has yet to be reported.

Testes are an important part of both the reproductive system and the endocrine system. The testes act to produce sperm and to produce androgens, primarily testosterone in the *Legdig* cells. In addition, the hypothalamic-pituitary-testes (HPT) axis is critical in the development and regulation of male reproduction. Testes have also been proven to be the key target organ of BPA toxicity [12], [13].Thus, exploration of testicular metabolite variations after BPA exposure will provide us an important understanding of BPA reproductive toxicity.

**Figure 1:**
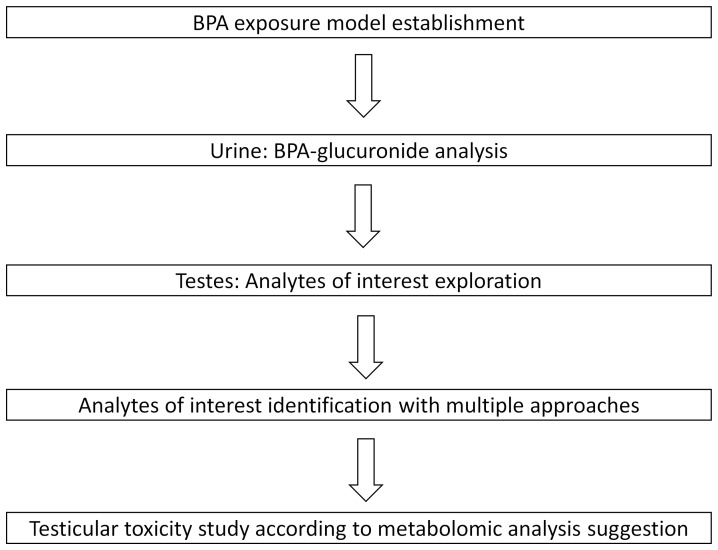
Workflow of this metabolomics study.

Here we used a non-targeted liquid chromatography-quadrupole time-of-flight (HPLC–QTOF) based metabolomic technique to study testicular toxicity of BPA using a model of adult rats exposed to BPA. This unbiased analysis allowed us a hypothesis-free exploration of endogenous compound’s metabolic perturbation cased by BPA exposure. The metabolomic analysis workflow is shown in Fig. 1.

## Materials and Methods

### Reagents

**Figure 2:**
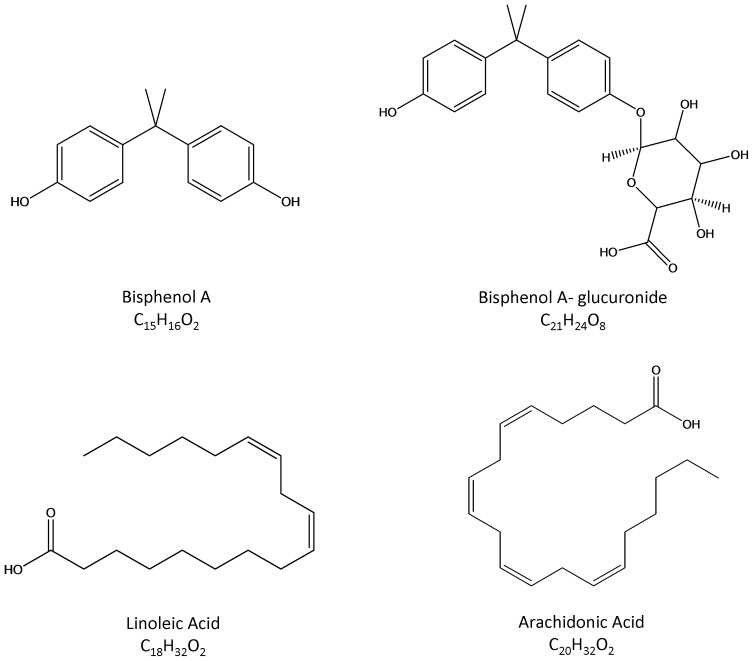
The structures of chemicals.

BPA and corn oil were purchased from Sigma Aldrich Laboratories, Inc. (St. Louis, MO, USA). BPA dosing solutions for rats were prepared in corn oil. Fresh solution of BPA in corn oil was made each week. Commercial assay kits for detection of superoxide dismutase (SOD) (SOD Detection Kit, Cat#A001-1), glutathione peroxidase (GSH-Px) (GSH-Px Detection Kit, Cat#A005) and catalase (CAT) (CAT Detection Kit, Cat#A007-2) were bought from Nanjing Jiancheng Biotech Ltd., China. Arachidonic acid (AA, purity≥99.0%) and linoleic acid (LA, purity≥99.0%) were purchased from Aladdin reagent company (Shanghai, China). Methanol was purchased from Merck Inc. (Darmstadt, Germany). Acetonitrile was purchased from Fisher Scientific. Water was purified with a Milli-Q purification system (Millipore, Bedford, MA). The structures of chemicals are shown in Fig. 2.

### Animals and Treatments

Male Sprague-Dawley rats were purchased from Slaccas (Slaccas Laboratory Animal, Shanghai, China). Twelve male Sprague-Dawley rats (180–200 g; 6–8 weeks) were housed under controlled humidity (40–60%) and temperature (20–24°C) with a 12 h light/dark cycle, and were randomized into two groups (six rats per group). Animals had free access to food and water. The animals were acclimated to the laboratory for 1 week prior to the start of the experiments. The rats in non-exposed group and exposed group were given daily gavage administration 0.5 mL of corn oil containing 0, 50 mg/kg BPA for 8 weeks, respectively (6 days/week). Using this gavage method, none of the rats died, and no injuries in the stomach, esophagus or trachea were found. After the last treatment, urine was collected using a metabolic cage that was placed under an ice bath so as to avoid the degradation of metabolites and urine was stored at −30°C. Several testes sections from each rat were placed into cryo-vials. After snap freezing in liquid nitrogen, they were preserved at −80°C. This study was carried out strictly in accordance with international standards on animal welfare and the guidelines of the Institute for Laboratory Animal Research of Nanjing Medical University. The protocol was approved by the Committee on the Ethics of Animal Experiments of Nanjing Medical University (Permit Number: BK2006576). All efforts were made to minimize animal suffering and to reduce the number of animals used.

### Metabolomic Analysis

The testes sample preparation procedure for LC-QTOF analysis was according to previous report with minor modification [14]. Approximately 25 mg of testes was homogenized in 1 mL water using a tissue disintegrator and an ultrasonic cell disruptor. Proteins were precipitated from homogenized tissue using 3 mL methanol and then centrifuged at 12000 g at 4°C for 15 min. For urine samples, a 1 mL sample of urine was mixed with 0.2 mL 1% formic acid in water and 4 mL methanol for protein precipitation and then centrifuged at 12000 g at 4°C for 15 min.

All supernatant from testes and urine precipitated extract was transferred and then dried in a vacuum. The dried supernatant extract was reconstituted with 200 µL 0.1% formic acid in 50/50 acetonitrile/water (V/V). All reconstitutions were centrifuged at 12000 g at 4°C for 10 min before LC injection. To avoid potential contamination, a blank sample for testes, which was prepared without adding testes, was analyzed in parallel with testicular samples.

LC-QTOF was carried out using an Agilent (1260) HPLC (Agilent Corporation, USA) with an electrospray ionization source (ESI) coupled to a micrOTOF-Q II mass spectrometer (Bruker Daltonics Inc., Billerica, MA). The mobile phase consisted of 0.1% formic acid in water (solvent A) and 0.1% formic acid in acetonitrile (solvent B). The reconstitutions of urine and testes were loaded onto a ZORBAX SB-C18 Column (2.1×150 mm; Agilent) maintained at 30°C, and gradient eluted (for urine: 2% B, two minutes; 2%−50% B, thirteen minutes; 50%−80% B, three minutes; 80%−95% B, five minutes; 95% B, five minutes; 2% B, thirteen minutes; for testes: 5% B, two minutes; 5%−50% B, six minutes; 50%−80% B, nine minutes; 80%−95% B, five minutes; 95% B, five minutes; 5% B, thirteen minutes) directly into the mass spectrometer at a flow rate of 0.3 mL/min. The injection volume was 10 µL.

The parameters for QTOF were as follows: ESI source positive and negative full scan mode, capillary voltage −4500 V for positive ion scan and 3500 V for negative ion scan, offset for end plat 500 V, capillary exit voltage 120–160 V, Nebulizer 1 bar, Dry Gas 6 L/min, Dry Heater 220°C. Mass range collected for testes: 50–1500 *m/z* for positive ion scan, 100–1500 *m/z* for negative ion scan; for urine: 50–1000 *m/z* for both positive and negative ion scan. Solution of sodium trifluoroacetate was used for the external calibration. The injection order of samples from the different treatment groups was completely randomized.

Bucket tables for statistical comparison of urinary BPA-glucuronide and unknown testicular analytes levels between groups were generated by ProfileAnalysis 2.0 software (Bruker Daltonics Corporation) using FindMolecularFeatures (FMF) compounds and advanced bucketing (Bucket filter 75%; Pareto scaling).

To chemically define the structures of the analytes selected for further investigation, multiple approaches were used. First, by using SmartFormula software (Bruker Corporation), possible molecular formulas of metabolites were provided. This software combines accurate mass (<4 mDa) and isotopic patterns (<25 mSigma) for enhanced confidence of molecular formulas identification. Second, potential biomarkers in testes were preliminarily identified by detailed information comparison with Human Metabolome Database (HMDB, http://www.hmdb.ca/). This comparison matched experimentally derived data to the library database of molecular weight, molecular formulas and related biological information created from known chemical entities. Finally, potential biomarker structure identification was confirmed by comparing retention times with standards using the same LC separation condition.

### Detection of Testicular Antioxidant Enzymes

SOD, GSH-Px and CAT levels in testes were assayed using commercial spectrophotometric kits according to the manufacturer's protocol (Jiancheng Biotech Ltd., Nanjing, China). Briefly, SOD was assayed based on its ability to decrease the oxidation of hydroxylamine to form a red product. GSH-Px was measured by detecting absorbance of a yellow product formed in the reaction between reduced glutathione and dithiobisnitrobenzoic acid. CAT was determined based on the fact that ammonium molybdate could rapidly terminate the degradation reaction of hydrogen peroxide catalyzed by CAT and react with the residual hydrogen peroxide to generate a yellow product. Total protein concentration in testicular homogenate samples was determined using the Coomassie blue method.

### Statistical Analyses

Urinary BPA-glucuronide, unknown testicular analytes and antioxidant enzymes levels between groups were compared with a two-sided Wilcoxon rank-sum test (*p*-values acquired from permutation). The correlation between levels of unknown analytes was tested with Spearman correlation test. Statistical significance was assumed to *p*<0.05.

## Results

### Urinary BPA-glucuronide Analysis

**Figure 3:**
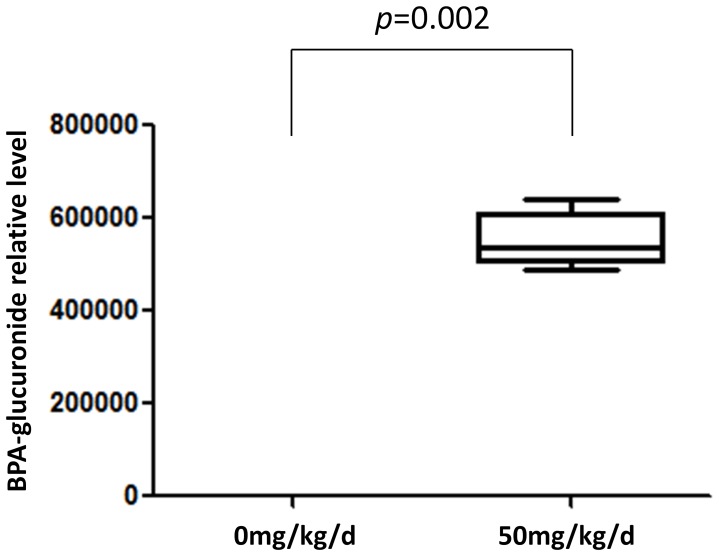
Difference of BPA-glucuronide relative levels between non-exposed and exposed groups. Box plot of the median and range of BPA-glucuronide relative levels. The top and bottom of the box represent the seventy-fifth and twenty-fifth percentile. The whiskers indicate the maximum and minimum points.

This unbiased metabolomics system showed good efficiency in finding and monitoring known BPA-glucuronide in rat urine, which is a major urinary metabolite of BPA and has been commonly monitored to reflect BPA internal exposure levels [15], [16], [17]. It was reported that BPA-glucuronide in urine was determined in negative ion model [15], [17]. This compound was found as [2M-H]^−^ in negative ion model in this study. The experimental derived data of BPA-glucuronide is shown in Table 1. We found BPA-glucuronide level increased dramatically in the BPA exposed group (*p* = 0.002) (Fig. 3). No detectable contamination of BPA in the non-exposed group was observed. By comparing the internal exposure level, we found that this BPA exposure model was successful and reasonable. Moreover, this metabolomic analysis for identifying and comparing known analyte validated the availability of this non-targeted technique in the exploration and identification of unknown testicular biomarkers.

**Table 1:** BPA-glucuronide experimentally derived data.

*m/z*	Molecularion	Retention time(min)	Predicted chemicalformula^a^	BPA-glucuronideformula	Error(mDa)
807.2879	[2M-H]^−^	20.6	C_21_H_24_O_8_	C_21_H_24_O_8_	−0.9

a Predicted with SmartFormula software with mSigma = 5.0.

### Testicular Potential Biomarkers Finding

**Figure 4:**
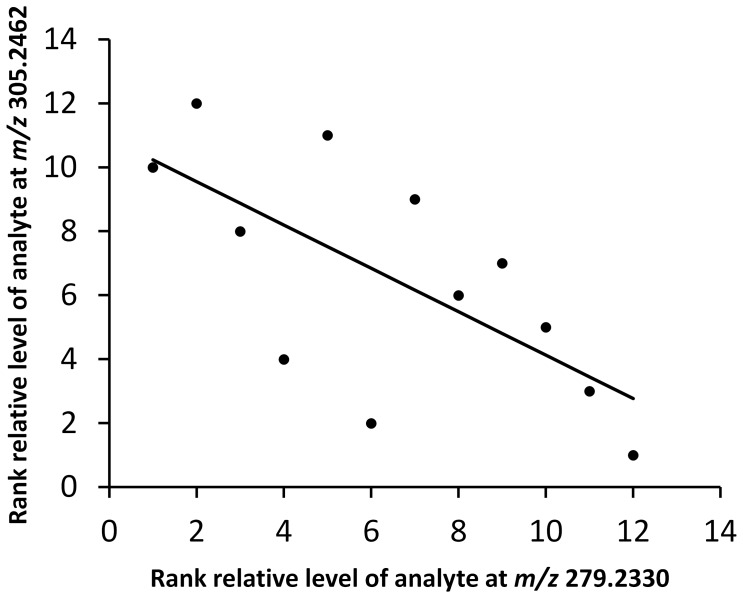
Spearman rank correlation between rank relative level of analytes at *m/z* 279.2330 and rank relative level of analytes *at m/z* 305.2462 (*r_s_* = −**0.6783,** ***p*** **= 0.0153,** ***N*** **= 12).**

**Figure 5.The:**
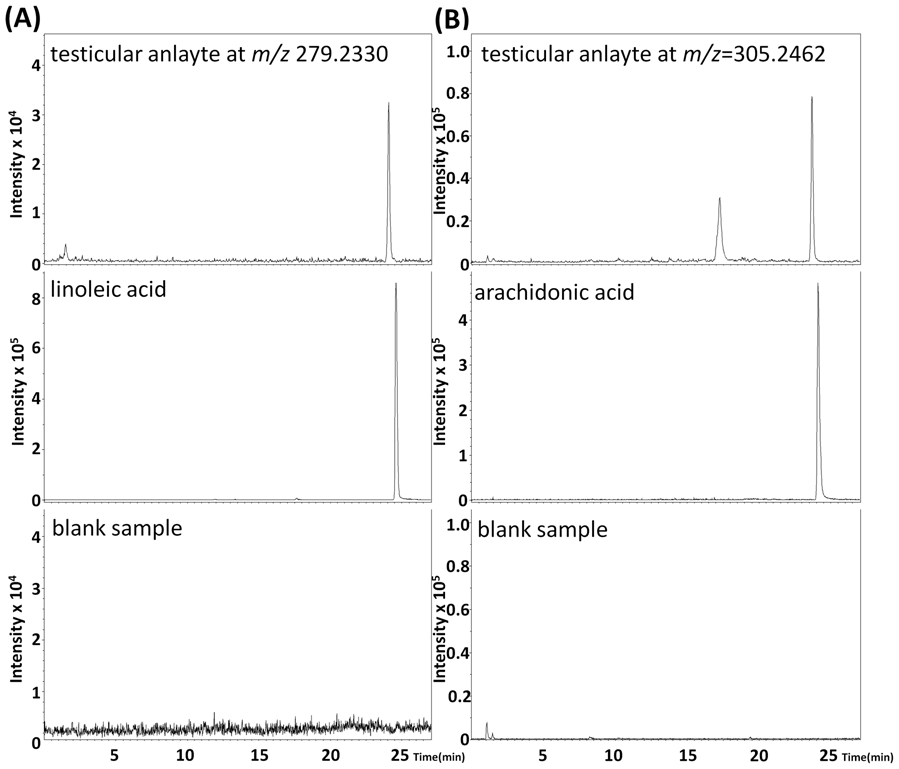
The extracted ion chromatograms. A.Testicular analytes at m/z 279.2330, LA and blank sample. B. Testicular analytes at *m/z* 305.2462, AA and blank sample.

As the testicular compounds screened were hypothesis-free, the structural identity of the small molecules found from the unbiased metabolomic analysis was not known. Since large amounts of endogenous compounds that might track with BPA toxicity were our concern, thus we initially removed the candidate compounds characterized by *m/z* showing data zero. Then, we removed the candidate compounds did not show an apparent chromatographic peak in the extracted chromatograms. Next, for improving structure identification confidence, we removed those candidate compounds when their formula predictions had mSigma>25 or accurate mass >4 mDa with SmartFormula software. Subsequently, we compared information of the remaining potential analytes with data in HMDB. Finally, we focused on those with *m/z* 279.2330 in negative ion mode and 305.2462 in positive ion mode. Those analytes with *m/z* 279.2330 and 305.2462 showed a significant difference between non-exposed and exposed groups (*p* = 0.0148 for *m/z* 279.2330; *p* = 0.0042 for 305.2462). Moreover, these two analytes demonstrated significant (*r_s_* = −0.6783, *p* = 0.0153, *N* = 12) negative correlation among each sample, suggesting a potential relationship via a common biochemical pathway and an important biological significance (Fig. 4). By examining the blank sample, we found no contamination of the two analytes (Fig. 5).

### Testicular Analytes Structural Identification

By using SmartFormula software, the analyte with *m/z* 279.2330 was predicted as C_18_H_32_O_2_, and the analyte with *m/z* 305.2462 was predicted as C_20_H_32_O_2_. We used HMDB to initially identify the two analytes. After structurally comparing molecular weight and molecular formulas, the analyte with *m/z* 279.2330 was initially identified as LA, and *m/z* 305.2462 was initially identified as AA (Fig. 1).The structure prediction was also supported by biological significance: (i) LA and AA are abundant in testes [18]; (ii) AA is biosynthesized from LA via Δ6 desaturation and subsequent 2-carbon elongation and Δ5 desaturation [19].

To further identify the two analytes, retention times of LA and AA standards were compared with that of corresponding unknown analytes using the same LC/MS condition, respectively. We found the two unknown analytes showed the same retention time advance (0.5 min) when compared with corresponding standards (Fig. 5). So the retention time difference was due to systematic error. Thus we identified the two analytes were LA and AA. The experimentally derived data of the two potential biomarkers identification is shown in Table 2.

**Table 2:** The experimentally derived data of the two potential testicular biomarkers identification.

Chemical information		Experimentally derived data of testicular analytes					Experimentally derived data of standards	
Chemical	Chemical formula	*m/z*	Ion mode	Retentiontime (min)	Predicted chemical formula^a^	Error (mDa)	Error (mDa)	ΔRetention time (min)^b^
Linoleic Acid	C_18_H_32_O_2_	279.2330	Negative	24.1	C_18_H_32_O_2_	0.4	−1.8	−0.5
Arachidonic Acid	C_20_H_32_O_2_	305.2462	Positive	23.6	C_20_H_32_O_2_	1.0	1.1	−0.5

a Predicted with SmartFormula software (mSigma = 5.1 for analyte at *m/z* 279.2330; mSigma = 3.4 for analyte at *m/z* 305.2462).

b Retention time of unknown analyte minus retention time of standard.

### BPA Exposure Altered Polyunsaturated Fatty Acid Composition in Testes

**Figure 6:**
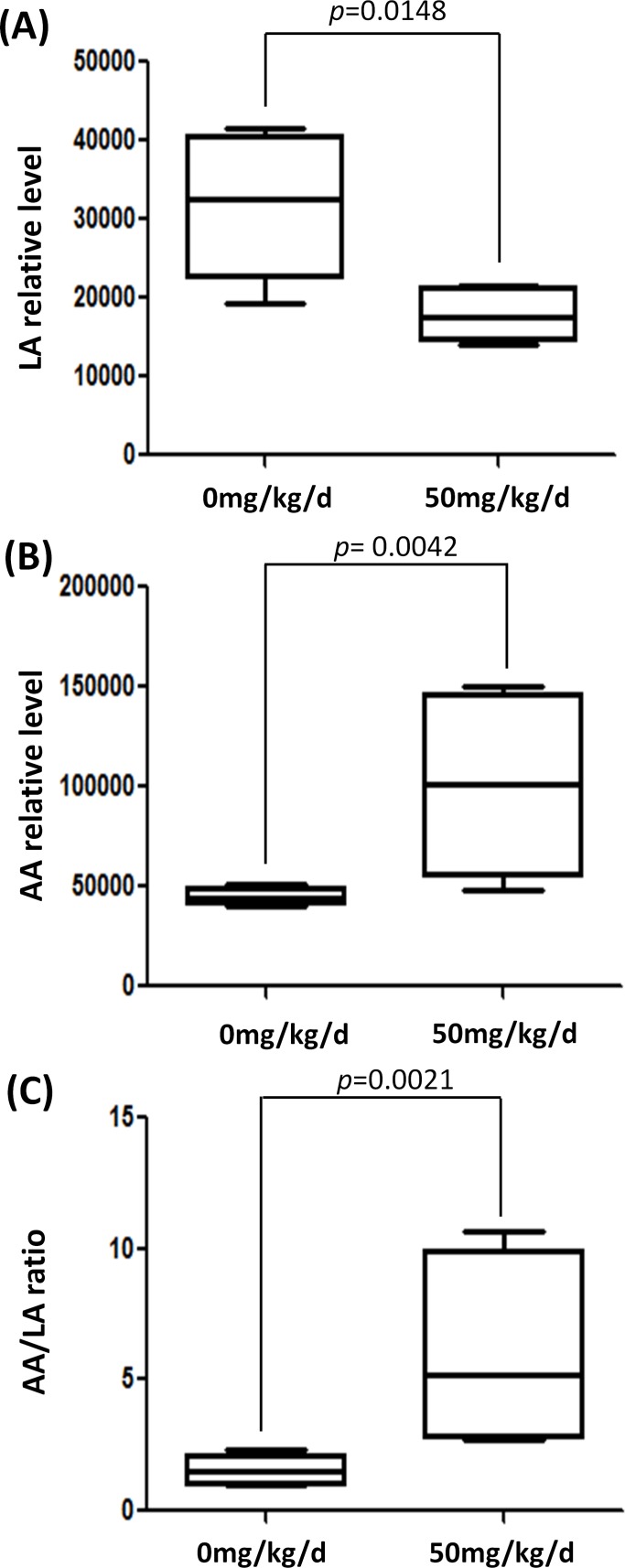
Difference of testicular n-6 fatty acids relative levels between non-exposed and exposed groups. A. Box plot of the median and range of LA relative levels. B. Box plot of the median and range of AA relative levels. C. Box plot of the median and range of AA/LA ratio. The top and bottom of the box represent the seventy-fifth and twenty-fifth percentile. The whiskers indicate the maximum and minimum points.

The testicular LA and AA relative levels between BPA exposed and non-exposed groups are shown in Fig. 6. We found testicular LA was decreased significantly in the 50 mg/kg/d BPA exposed group (*p* = 0.0148), while AA was contrary (*p* = 0.0042) (Fig. 6 A,B). Since LA is a precursor to AA [19], these changes suggested enhanced conversion of LA to AA in testes. Thus, we calculated the AA relative levels/LA relative levels ratio of each rat. Accordingly, this ratio was increased significantly in the BPA exposed group (*p* = 0.0021) (Fig. 6 C).

### BPA Exposure Decreased Testicular Antioxidant Enzymes

**Figure 7:**
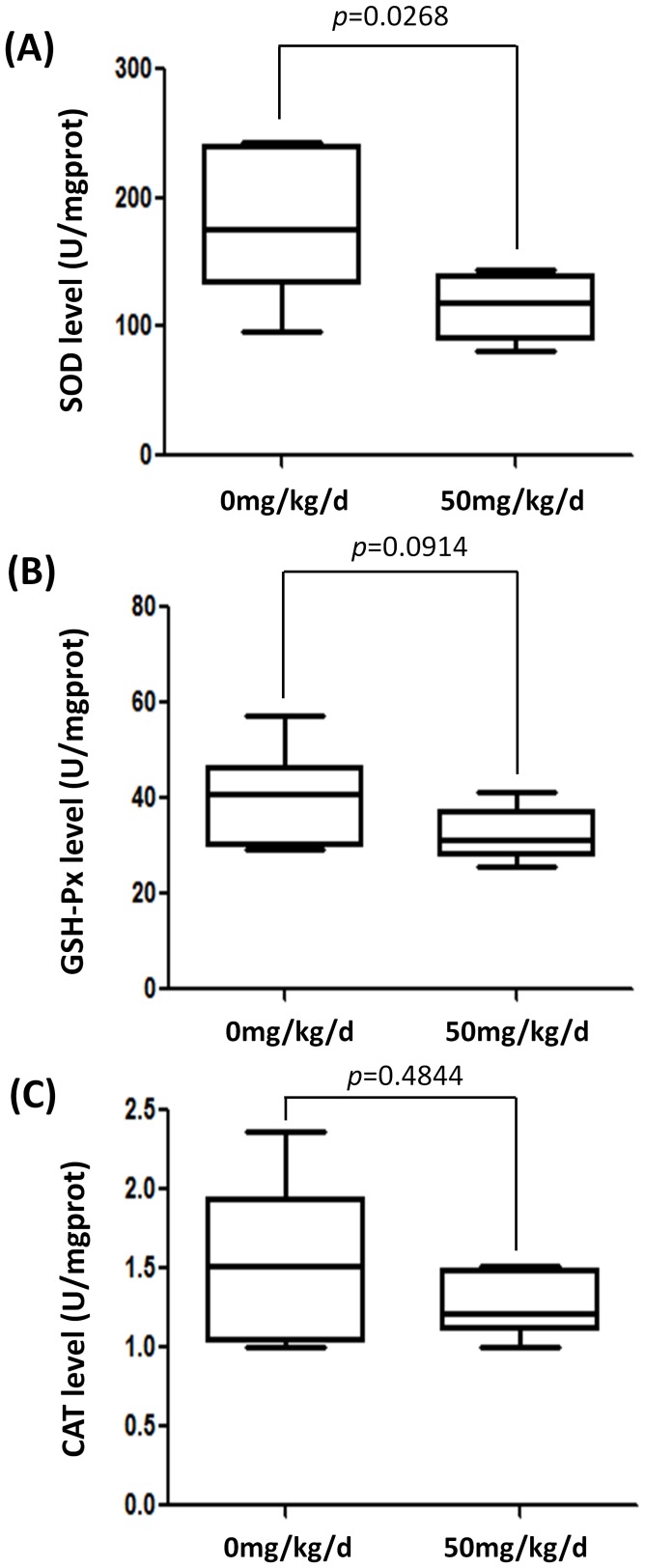
Difference of testicular antioxidant enzymes levels between non-exposed and exposed groups. A. Box plot of the median and range of SOD level (U/mgprot). B. Box plot of the median and range of GSH-Px level (U/mgprot). C. Box plot of the median and range of CAT level (U/mgprot). The top and bottom of the box represent the seventy-fifth and twenty-fifth percentile. The whiskers indicate the ma ximum and minimum points.

Polyunsaturated fatty acid (PUFA) is very susceptible to peroxidation [20], and AA is a accepted better substrate for lipid peroxidation (LP) than LA [21], [22]. AA (20∶4 n-6) is a compound of important potent bioactivity. Besides acting as a membrane component and providing energy storage, AA also acts as a signal molecule in regulating steroidogenesis in *Leydig* cells [23]. Previous work indicated that an elevated process of LA conversion to AA might act as a regulatory system to prevent depletion of AA during periods of oxidative stress owing to the important physiological functions of this fatty acid in testes [24]. Similarly, increased AA/LA ratio, AA level and corresponding ascending oxidative stress were found in chick liver, serum and erythrocyte membranes after lead exposure [19]. Thus, we speculated that the PUFA composition alterations indicated testicular oxidative stress. We assayed SOD, CAT and GSH-Px levels in testes, and found significant decreased SOD levels in exposed group (*p* = 0.0268) (Fig. 7A). The GSH-Px and CAT also showed a decreasing trend in the 50 mg/kg/d treated group (Fig. 7 B,C). The decrease of GSH-Px was suggestive, borderline-significant (*p* = 0.0914) (Fig. 6 B). These data were consistent with the hypothesis that testicular oxidative stress was increased in the BPA exposed group.

## Discussion

In this study, we validated an unbiased metabolomics system and a rat model of exposure to BPA through finding and analyzing known urinary BPA metabolite, and used this metabolomics system to discover unknown metabolite changes in testes. With multiple identification approaches, we identified AA and LA which might be potential biomarkers of BPA exposure. Decreased levels of LA and increased levels of AA as well as AA/LA ratio were found in BPA exposed group. Because this kind of varied fatty acid composition might biologically adapt to oxidative stress [24], we predicted that oxidative stress occurred in the testes, and found supporting evidence that testicular antioxidant enzymes declined. Additionally, in this rat model, sperm numbers decreased in BPA treated group when compared with that of non-exposed group (unpublished data).

Lipids are abundant in testicles. Phospholipid is the largest component, while triglyceride is present in smaller quantities [18]. LA (18∶2 n-6) is the major PUFA in vegetable oils and is a metabolic precursor to AA (20∶4 n-6) [19]. AA mainly exists in phospholipid and LA mainly exists in phospholipid and triglyceride [18]. Lipids play a critical role in membrane structure and function, energy storage and cell signaling [25]. A Previous report has indicated that LA family PUFA plays an important role in testicular function [18]. Moreover, it is reported that product-to-precursor ratios can strengthen the association signal and provide new information about possible metabolic pathways in metabolomics study [10]. The use of product-to-precursor ratio (eg, AA/LA ratio) as a surrogate measure to estimate desaturase activity is well established [26]. In this study we calculated the AA/LA ratio, and found this ratio was elevated and, therefore, BPA exposure may increase desaturase activity and enhance the metabolic process of LA conversion to AA in testes. Accordingly, a previous study found that testosterone treated *Sertoli* cell showed significant increase of LA (18∶2 n-6), decrease of AA (20∶4 n-6), decrease in AA/LA ratio and drop in desaturase activities [27]. PUFA composition change observed in this metabolomics study was consistent with decreased spermatogenesis in this rat model. Therefore, testicular AA and LA alteration might be involved in BPA testicular toxicity.

Oxidant/antioxidant imbalance in the testes may induce oxidative stress and thereby hamper testicular function [28]. Building on implication from metabolomics study, we further assayed testicular antioxidant enzymes, and found a significantly decreased level of SOD in the exposed group, as well as a decreasing trend in GSH-Px and CAT. Moreover, despite AA and LA as substrate for LP [19], [21], AA has also been shown as a better oxidative stress inducer than LA [29]. Combing these findings, testicular oxidative stress might occur in BPA treated rats, which supports our previous hypothesis based on metabolomics data. Testicular oxidative stress induced by BPA exposure was also reported in a recent published report [12]. PUFA which is apt to be oxidized is abundant in testes [18], [20], and oxidative stress could cause both membrane lipid peroxidation and DNA fragmentation in testes [30]. It is reported that spermatogenesis [31] and *Leydig* cell steroidogenisis [32] are both vulnerable to oxidative stress. Clinical studies have demonstrated that male infertility patients showed higher oxidative stress [33] and related decreased SOD and GSH-Px levels [34]. Additionally, oxidative stress and disturbed equilibrium of oxidant/antioxidant has been suggested as a major mechanism of reproductive toxicity [35]. A plausible explanation of decreased spermatogenesis of the rat model in present study is oxidant/antioxidant imbalance, which is widely supported by population and animal studies [36], [37].

Metabolomic analysis of urinary known analyte showed great amounts of BPA-glucuronide excreted in rat urine. This finding agreed with a previous report [16]. To our knowledge, although BPA-exposed animal studies were widely reported, few studies assayed internal BPA exposure level [38]. By comparing the urinary BPA-glucuronide levels, we proved that this rat exposure model was non-contaminated and successful. BPA internal exposure level monitoring in BPA exposure model appears necessary, because BPA is widely used and, therefore, whether contamination occurs during animal feeding process should be examined [4].

Within the toxicology community, although metabolomics has been widely used in exploring renal [14], [39] and hepatic [11], [40] toxicity of chemicals, the use of metabolomics in the study which focuses on reproductive toxicity is still an intriguing new field. The testicle is the primary gonad of the reproduction and endocrine systems in male animals, and metabolomic analysis of this organ would provide direct understanding of metabolites change in reproduction systems. This metabolomics study showed the availability of metabolomic analysis of testes in finding biomarkers and providing mechanistic insights into reproductive toxicity. It has been widely reported that exposure of rats to BPA at 50 mg/kg/d caused adverse effects on reproduction [38].For we aimed to explore the BPA basic toxicity using metabolomic analysis, this study only used one dose group (50 mg/kg/d) which is the currently accepted lowest observed adverse effect level (LOAEL) [1]. Since testicular toxicity in lower doses was presented in previous studies [12], [13], [38], future work needs to study lower dose groups to provide more metabolomics understandings of BPA reproductive toxicity.

In conclusion, using metabolomic analysis with LC-QTOF, we found and structurally identified two potential biomarkers, LA and AA.The BPA-induced testicular toxicity showed decreased LA levels, as well as increased AA levels and AA/LA ratio. Based on the suggestion of fatty acid composition variation, an imbalance of the antioxidant enzyme system was found. This study highlights the application of metabolomics to the discovery of chemical reproductive toxicity.

## References

[1] United States Environmental Protection Agency (2010) Bisphenol A Action Plan (CASRN 80-05-7).Available: http://www.epa.gov/oppt/existingchemicals/pubs/actionplans/bpa_action_plan.pdf.Accessed 2012 Feb 13.

[2] Vandenberg LN, Chahoud I, Heindel JJ, Padmanabhan V, Paumgartten FJ, et al (2010) Urinary, circulating, and tissue biomonitoring studies indicate widespread exposure to bisphenol A. Environ Health Perspect. 118: 1055–1070. 10.1289/ehp.0901716 PMC2920080 20338858

[3] Diamanti-Kandarakis E, Bourguignon JP, Giudice LC, Hauser R, Prins GS, et al (2009) Endocrine-disrupting chemicals: an Endocrine Society scientific statement. Endocr Rev 30: 293–342. 19502515 10.1210/er.2009-0002 PMC2726844

[4] Vandenberg LN, Hauser R, Marcus M, Olea N, Welshons WV (2007) Human exposure to bisphenol A (BPA). Reprod Toxicol 24: 139–177. 17825522 10.1016/j.reprotox.2007.07.010

[5] Calafat AM, Ye X, Wong LY, Reidy JA, Needham LL (2008) Exposure of the U.S. population to bisphenol A and 4-tertiary-octylphenol: 2003–2004. Environ Health Perspect 116: 39–44. 18197297 10.1289/ehp.10753 PMC2199288

[6] Li DK, Zhou Z, Miao M, He Y, Wang J, et al (2011) Urine bisphenol-A (BPA) level in relation to semen quality. Fertil Steril 95: 625–630. 21035116 10.1016/j.fertnstert.2010.09.026

[7] Meeker JD, Ehrlich S, Toth TL, Wright DL, Calafat AM, et al (2010) Semen quality and sperm DNA damage in relation to urinary bisphenol A among men from an infertility clinic. Reprod Toxicol 30: 532–539. 20656017 10.1016/j.reprotox.2010.07.005 PMC2993767

[8] Li YJ, Song TB, Cai YY, Zhou JS, Song X, et al (2009) Bisphenol A exposure induces apoptosis and upregulation of Fas/FasL and caspase-3 expression in the testes of mice. Toxicol Sci 108: 427–436. 19193734 10.1093/toxsci/kfp024

[9] Izumi Y, Yamaguchi K, Ishikawa T, Ando M, Chiba K, et al (2011) Molecular changes induced by bisphenol-A in rat Sertoli cell culture. Syst Biol Reprod Med 57: 228–232. 21574817 10.3109/19396368.2011.574248

[10] Suhre K, Shin SY, Petersen AK, Mohney RP, Meredith D, et al (2011) Human metabolic individuality in biomedical and pharmaceutical research. Nature 477: 54–60. 21886157 10.1038/nature10354 PMC3832838

[11] Parman T, Bunin DI, Ng HH, McDunn JE, Wulff JE, et al (2011) Toxicogenomics and metabolomics of pentamethylchromanol (PMCol)-induced hepatotoxicity. Toxicol Sci 124: 487–501. 21920950 10.1093/toxsci/kfr238 PMC3247806

[12] D'Cruz SC, Jubendradass R, Mathur PP (2012) Bisphenol A Induces Oxidative Stress and Decreases Levels of Insulin Receptor Substrate 2 and Glucose Transporter 8 in Rat Testis. Reprod Sci 19: 163–172. 22101236 10.1177/1933719111415547

[13] D'Cruz SC, Jubendradass R, Jayakanthan M, Rani SJ, Mathur PP (2012) Bisphenol A impairs insulin signaling and glucose homeostasis and decreases steroidogenesis in rat testis: An in vivo and in silico study. Food Chem Toxicol 50: 1124–1133. 22142692 10.1016/j.fct.2011.11.041

[14] Boudonck KJ, Mitchell MW, Német L, Keresztes L, Nyska A, et al (2009) Discovery of metabolomics biomarkers for early detection of nephrotoxicity. Toxicol Pathol 37: 280–292. 19380839 10.1177/0192623309332992

[15] Ye X, Kuklenyik Z, Needham LL, Calafat AM (2005) Quantification of urinary conjugates of bisphenol A, 2,5-dichlorophenol, and 2-hydroxy-4-methoxybenzophenone in humans by online solid phase extraction-high performance liquid chromatography-tandem mass spectrometry. Anal Bioanal Chem 383: 638–644. 16132150 10.1007/s00216-005-0019-4

[16] Pottenger LH, Domoradzki JY, Markham DA, Hansen SC, Cagen SZ, et al (2000) The relative bioavailability and metabolism of bisphenol A in rats is dependent upon the route of administration. Toxicol Sci 54: 3–18. 10746927 10.1093/toxsci/54.1.3

[17] Völkel W, Bittner N, Dekant W (2005) Quantitation of bisphenol A and bisphenol A glucuronide in biological samples by high performance liquid chromatography-tandem mass spectrometry. Drug Metab Dispos 33: 1748–1757. 16103135 10.1124/dmd.105.005454

[18] Davis JT, Bridges RB, Coniglio JG (1966) Changes in lipid composition of the maturing rat testis. Biochem J 98: 342–346. 5949372 10.1042/bj0980342 PMC1264837

[19] Lawton LJ, Donaldson WE (1991) Lead-induced tissue fatty acid alterations and lipid peroxidation. Biol Trace Elem Res 28: 83–97. 1709034 10.1007/BF02863075

[20] Jordan RA, Schenkman JB (1982) Relationship between malondialdehyde production and arachidonate consumption during NADPH-supported microsomal lipid peroxidation. Biochem Pharmacol 31: 1393–1400. 6807321 10.1016/0006-2952(82)90034-x

[21] Bruna E, Petit E, Beljean-Leymarie M, Huynh S, Nouvelot A (1989) Specific susceptibility of docosahexaenoic acid and eicosapentaenoic acid to peroxidation in aqueous solution. Lipids 24: 970–975.

[22] Lokesh BR, Mathur SN, Spector AA (1981) Effect of fatty acid saturation on NADPH-dependent lipid peroxidation in rat liver microsomes. J Lipid Res 22: 905–915. 6792310

[23] Wang X, Walsh LP, Reinhart AJ, Stocco DM (2000) The role of arachidonic acid in steroidogenesis and steroidogenic acute regulatory (StAR) gene and protein expression. J Biol Chem 275: 20204–20209. 10777507 10.1074/jbc.M003113200

[24] Burczynski JM, Southard SJ, Hayes JR, Longhurst PA, Colby HD (2001) Changes in mitochondrial and microsomal lipid peroxidation and fatty acid profiles in adrenal glands, testes, and livers from alpha-tocopherol-deficient rats. Free Radic Biol Med 30: 1029–1035. 11316583 10.1016/s0891-5849(01)00497-x

[25] Oresic M, Hänninen VA, Vidal-Puig A (2008) Lipidomics: a new window to biomedical frontiers. Trends Biotechnol 26: 647–652. 18951641 10.1016/j.tibtech.2008.09.001

[26] Vessby B, Gustafsson IB, Tengblad S, Boberg M, Andersson A (2002) Desaturation and elongation of Fatty acids and insulin action. Ann N Y Acad Sci 967: 183–195. 12079847 10.1111/j.1749-6632.2002.tb04275.x

[27] Hurtado de Catalfo GE, de Gómez Dumm IN (2005) Influence of testosterone on polyunsaturated fatty acid biosynthesis in Sertoli cells in culture. Cell Biochem Funct 23: 175–180. 15376235 10.1002/cbf.1135

[28] Saradha B, Mathur PP (2006) Effect of environmental contaminants on male reproduction. Environ Toxicol Pharmacol 21: 34–41. 21783636 10.1016/j.etap.2005.06.004

[29] Aitken RJ, Wingate JK, De Iuliis GN, Koppers AJ, McLaughlin EA (2006) Cis-unsaturated fatty acids stimulate reactive oxygen species generation and lipid peroxidation in human spermatozoa. J Clin Endocrinol Metab 91: 4154–4163. 16895947 10.1210/jc.2006-1309

[30] Aitken RJ, Roman SD (2008) Antioxidant systems and oxidative stress in the testes. Adv Exp Med Biol 636: 154–171. 19856167 10.1007/978-0-387-09597-4_9

[31] Naughton CK, Nangia AK, Agarwal A (2001) Pathophysiology of varicoceles in male infertility. Hum Reprod Update 7: 473–481. 11556494 10.1093/humupd/7.5.473

[32] Hales DB, Allen JA, Shankara T, Janus P, Buck S, et al (2005) Mitochondrial function in Leydig cell steroidogenesis. Ann N Y Acad Sci 1061: 120–134. 16469751 10.1196/annals.1336.014

[33] Sikka SC (2001) Relative impact of oxidative stress on male reproductive function. Curr Med Chem 8: 851–862. 11375755 10.2174/0929867013373039

[34] Dandekar SP, Nadkarni GD, Kulkarni VS, Punekar S (2002) Lipid peroxidation and antioxidant enzymes in male infertility. J Postgrad Med 48: 186–189. 12432192

[35] Mathur PP, D'Cruz SC (2011) The effect of environmental contaminants on testicular function. Asian J Androl 13: 585–591. 21706039 10.1038/aja.2011.40 PMC3739630

[36] Appasamy M, Muttukrishna S, Pizzey AR, Ozturk O, Groome NP, et al (2007) Relationship between male reproductive hormones, sperm DNA damage and markers of oxidative stress in infertility. Reprod Biomed Online 14: 159–165. 17298717 10.1016/s1472-6483(10)60783-3

[37] Aly HA, Domènech O, Abdel-Naim AB (2009) Aroclor 1254 impairs spermatogenesis and induces oxidative stress in rat testicular mitochondria. Food Chem Toxicol 47: 1733–1738. 19306909 10.1016/j.fct.2009.03.019

[38] Richter CA, Birnbaum LS, Farabollini F, Newbold RR, Rubin BS, et al (2007) In vivo effects of bisphenol A in laboratory rodent studies. Reprod Toxicol 24: 199–224. 17683900 10.1016/j.reprotox.2007.06.004 PMC2151845

[39] Sieber M, Hoffmann D, Adler M, Vaidya VS, Clement M, et al (2009) Comparative analysis of novel noninvasive renal biomarkers and metabonomic changes in a rat model of gentamicin nephrotoxicity. Toxicol Sci 109: 336–349. 19349640 10.1093/toxsci/kfp070 PMC4830225

[40] McBurney RN, Hines WM, Von Tungeln LS, Schnackenberg LK, Beger RD, et al (2009) The liver toxicity biomarker study: phase I design and preliminary results. Toxicol Pathol 37: 52–64. 19171931 10.1177/0192623308329287

